# Effects of Different Photoperiods on Growth Performance, Glucose Metabolism, Acetylcholine, and Its Relative Acetylcholine Receptor Modulation in Broiler Chickens

**DOI:** 10.3390/ani14203003

**Published:** 2024-10-17

**Authors:** Miao Yu, Mengjie Xu, Guangju Wang, Jinghai Feng, Minhong Zhang

**Affiliations:** 1 State Key Laboratory of Animal Nutrition and Feeding, Institute of Animal Sciences, Chinese Academy of Agricultural Sciences, Haidian, Beijing 100193, China; 82101211223@caas.cn (M.Y.); 82101215394@caas.cn (M.X.); guangju.wang@wur.nl (G.W.); fengjinghai@caas.cn (J.F.); 2 Adaptation Physiology Group, Wageningen University and Research, 6708 PB Wageningen, The Netherlands

**Keywords:** photoperiods, growth performance, acetylcholine, M3 muscarinic acetylcholine receptor, glucose metabolism, broiler

## Abstract

**Simple Summary:**

Long photoperiods are commonly used in modern intensive broiler chicken farming, which are crucial environmental factors in the growth and health of broilers. Through different photoperiods experiments, we found that extending the photoperiods could promote the growth rate of broilers while reducing the feed efficiency, inhibiting acetylcholine and its relative-acetylcholine receptor expression, and inducing glucose metabolism disorders in broilers. In addition, these findings have important implications for future research on the role of glucose metabolism, acetylcholine, and its relative acetylcholine receptors in regulating the health of broilers underlying extending the photoperiods.

**Abstract:**

Photoperiods are crucial environmental factors in the growth and health of modern intensive broiler chicken production. To date, the effects of different photoperiods on glucose metabolism, acetylcholine (ACh), and its relative acetylcholine receptor modulation in broilers remain elusive. Herein, we aimed to identify the effects of different photoperiods on regulating glucose metabolism, ACh, nicotinic acetylcholine receptor alpha 4 (α4 nAChR) mRNA, and M3 muscarinic acetylcholine receptor (M3 mAChR) modulation in broilers. A total of 216 healthy 5-day-old Arbor Acres (AA) male broilers was randomly assigned to 12L:12D, 18L:6D, and 24L:0D photoperiods for 4 weeks. The results show that, compared with the 12L:12D photoperiod, the 18L:6D and 24L:0D photoperiods significantly increase the average daily gain (ADG) and average daily feed intake (ADFI) of broilers (*p* < 0.05). However, the feed efficiency (FE) of broilers significantly decreased in the 18L:6D and 24L:0D photoperiods (*p* < 0.05). Moreover, compared with the 12L:12D photoperiod, the ACh concentrations and α4 nAChR mRNA expression levels in the hypothalamus and medulla oblongata of broilers significantly increased (*p* < 0.05); M3 mAChR mRNA expression levels in cecum significantly reduced in the 18L:6D photoperiod and the 24L:0D photoperiod (*p* < 0.05). Compared with the 12L:12D photoperiod, the serum glucose (GLU), serum insulin (INS), serum triglyceride (TG) levels, and homeostasis model assessment of insulin resistance (HOMA-IR) of broilers significantly enhanced in the 18L:6D and 24L:0D photoperiods (*p* < 0.05). Our results indicate that extending the photoperiod can promote the growth rate, ACh expression, and α4 nAChR mRNA expression of broilers while reducing the feed efficiency, inhibiting M3 mAChR mRNA expression, and inducing glucose metabolism disorders in broilers.

## 1. Introduction

The duration of light is a significant environmental factor in the modern broiler industry [1]. Long photoperiods, commonly used in the modern and intensive broiler industry, play a crucial role in maximizing production efficiency by improving feed intake and production performance [2]. Long photoperiods have been shown to significantly improve the feed intake and body weight of broilers [3,4,5]. Moreover, the manipulation of different photoperiods is also a critical environmental factor affecting the health and welfare of broilers. However, there are some studies in the literature regarding the negative impacts of inappropriate light duration, especially long light duration, on physiological stress, leg bone health, and behavioral patterns in broilers [3,4,5]. Indeed, it has been shown that the higher mortality of adult broilers is related to some physiological diseases caused by rapid growth rates under long photoperiods [6]. It has been also shown that metabolic disorders can be caused by rapid growth, a high nutrient intake, and a high metabolic rate in broilers [6].

In animal physiology, the secretion of acetylcholine (ACh) is regulated with light stimulation [7,8], which is the main neurotransmitter of the ACh signaling pathway. The ACh signaling pathway has a decisive intrinsic role in regulating the development and growth of birds, including the control of feeding behavior in neonatal broilers [9], egg-laying capacity in broilers [10], and prolactin-releasing factor secretion from the hypothalamus in broilers [11]. In the modern and intensive broiler industry, long photoperiods are widely used to maximize production efficiency by improving growth and production performance [2,3,4,5]. It has been reported that higher ACh concentrations could be released during light stimulation [7,8]. However, to date, there are currently no studies on the effects of different photoperiods on ACh and its relative acetylcholine receptor modulation in broilers.

Few studies have suggested that different photoperiods can regulate glucose metabolism homeostasis in birds and mammals. The photoperiod-dependent changes in the transcriptional regulation of carbohydrate metabolism was also observed in redheaded buntings (*Emberiza bruniceps*) exposed to the 13L:11D photoperiod and 8L:16D photoperiod [12]. Moreover, it was also reported that there were negative effects of the 18L:6D photoperiod on glucose metabolism and higher serum glucose concentrations and glucose intolerance in mice compared with the 12L:12D photoperiod [13]. Exposure to dim light at night can alter the daily rhythms of glucose metabolism in rats, which might lead to the development and progression of metabolic diseases [14]. Previous research has also found that artificial light is a high-risk factor for metabolic disorders, which can acutely decrease glucose tolerance in mice by the activation of the retina–ganglion cells–hypothalamic supraoptic nucleus axis [15]. Notably, the circadian clock system is essential for photoperiodic responses, which can also have a significant influence on glucose metabolism [16].Glucose metabolism displays an essential role that contributes to maintaining cellular energy metabolism and vital activities in broilers [17]. Yet, in broilers, the clear effects of different photoperiods on glucose metabolism are still unknown.

The ACh signaling pathway also plays an essential role in modulating glucose metabolism [18,19,20]. In rats, the functional decline in the cholinergic system induced by selective basal forebrain cholinergic neuron damage was associated with glucose hypometabolism [21]. In addition, the parasympathetic nervous system, as an ACh signaling pathway, performs its specialized functions through the primary neurotransmitter ACh, which is closely related to glucose metabolism. Previous studies have reported that the parasympathetic nerve system could relay glucoregulatory cues from the brain to peripheral tissues in mammals, particularly the pancreatic islet, to regulate glucose metabolism [22,23]. The activation of the parasympathetic system led to the suppression of hepatic gluconeogenesis and improvement of glucose tolerance in mice [24,25]. These findings encouraged us to identify the changes in glucose metabolism, ACh, and its relative acetylcholine receptor modulation of broilers induced by different photoperiods.

In this study, we aimed to identify the effects of different photoperiods on regulating serum glucose metabolism, ACh concentrations, α4 nAChR, and M3 mAChR modulation in broilers. We aspire to find the novel effects of long photoperiods on regulating physiological responses in broilers. The results of this study can provide us with new insights and serve as the basis for regulating glucose metabolism to improve the health of broilers exposed to long photoperiods.

## 2. Materials and Methods

The study was approved by the Institutional Ethics Committee of Experiment Animal Welfare and Ethics at the Institute of Animal Science of Chinese Academy of Agricultural Sciences (CAAS) (permit number: IAS 2022-117).

### 2.1. Birds and Experimental Design

A total of 5-day-old 216 AA male broilers with similar body weights (75 g ± 10) was randomly assigned to 3 photoperiod treatment groups with 6 replicates per treatment, with 12 broilers per replicate. The 3 groups were exposed to different photoperiods of 12L:12D (12 h light and 12 h dark, simulating a natural-lighting photoperiod), 18L:6D (18 h light and 6 h dark, a more suitable photoperiod), and 24L:0D (24 h light, a faster growth photoperiod) for 4 weeks. The light intensity was 15 lux. According to Zeitgeber time, 8:00 a.m. on the first day of the experiment was recorded as ZT0. The 12L:12D photoperiod had ZT0-ZT12 as the light period and ZT13-ZT24 as the dark period. The 18L:6D photoperiod had ZT0-ZT18 as the light period and ZT19-ZT24 as the dark period. The 24L:0D photoperiod had the ZT0-ZT24 as the light period. All groups received a standard corn–soybean-meal basal diet in 3 feeding programs (5–7 d, 8–21 d, and 22–33 d) (Table 1) [26], formulated according to AA broiler recommendations [27]. All broilers were housed in stainless, single-layer flat cages without roofs (0.82 m width × 0.70 m length × 0.60 m height), and 12 broilers per replicate were housed in 1 cage. All broilers were farmed in the artificial climate chambers (4.08 m × 2.88 m × 2.38 m) of the State Key Laboratory of Animal Nutrition and Feeding, Chinese Academy of Agricultural Sciences, and four cages were placed in one artificial climate chamber. Except for the photoperiods in the artificial climate chambers, other environmental parameters remained the same. The ambient temperature was determined according to the standard of the AA Broiler Feeding Management Manual [27]. The relative humidity was maintained at 60%. Broilers had free access to experimental diets and water.

### 2.2. Sample Collection

At the end of week 2 and week 4 in the trial period, 1 broiler from each replicate was selected with a body weight close to the average after 12 h of feed deprivation. All samples of broilers were collected at ZT1. Blood samples were obtained via vacuum blood collection tubes from the wing vein of the broilers. The serum was separated from blood using centrifugation at 3000 r/min for 10 min at 4 °C; the supernatant was collected and stored at −20 °C until it was used for the biochemical analysis. Then, the broilers from each group were killed using carbon dioxide (CO_2_); the CO_2_ concentration was 60%. We cut off the heads of the broilers, then longitudinally cut the skin and opened the skull to expose the brain. Tissue samples of the hypothalamus and medulla oblongata were obtained under a red light in a dark room, immediately collected and snap-frozen in liquid nitrogen, then kept in a −80 °C freezer for measurements of gene expression and ACh concentrations analysis. Tissue samples of the cecum (3 cm from the end of the cecum) were rinsed with sterile normal saline (NaCl 9 g/L) and immediately snap-frozen in liquid nitrogen, then kept in a −80 °C freezer for measurements of gene expression analysis.

### 2.3. Growth Performance

At the end of week 2 and week 4 in the trial period, the provided and residual feed amount and broiler body weight of each replicate were recorded. The average daily gain (ADG), average daily feed intake (ADFI), and feed efficiency (FE, FE = ADG/ADFI) were calculated.

### 2.4. Serum Glycolysis Metabolism Analysis

The enzymatic hexokinase endpoint method was used to measure the concentrations of serum glucose (GLU). Three parallels were made for each sample. The serum GLU assay was performed using the Hitachi 7600 automatic biochemical analyzer (Hitachi 7600, Hitachi Ltd., Tokyo, Japan), according to the commercial kit’s instructions (Beijing Leadman Biochemical Co., Ltd., Beijing, China).

The lipase/glycerokinase bichromatic end-point method was used to measure the concentrations of serum triglyceride (TG). Three parallels were made for each sample. The serum TG assay was performed using the Hitachi 7600 automatic biochemical analyzer (Hitachi 7600, Hitachi Ltd., Tokyo, Japan), according to the commercial kit’s instructions (Beijing Leadman Biochemical Co., Ltd., Beijing, China).

The radioimmunoassay method was used to measure the concentrations of serum insulin (INS). Three parallels were made for each sample. The serum INS assay was performed using the γ-type immune counter (Anhui Zhongke Zhongjia Scientific Instrument Co., Ltd., Hefei, China), according to the commercial kit’s instructions (Tianjin Xiehe Pharmaceutical Technology Co., Ltd., Tianjin, China).

The evaluated indicator of insulin resistance was the homeostasis model assessment of insulin resistance (HOMA-IR). To calculate the HOMA-IR, we used the following calculation formula: HOMA-IR = fasting blood glucose (FBG, mmol/L) × fasting serum insulin (FINS, mU/L) ÷ 22.5.

### 2.5. ACh Concentration Analyses of the Hypothalamus and Medulla Oblongata

The concentrations of ACh in the hypothalamus and medulla oblongata were measured using ELISA kits (A105-2-1, Nanjing Jiancheng Bioengineering Institute, Nanjing, China) by the Multiskan MK3 microplate reader (Thermo Fisher Scientific, Waltham, MA, USA), following the instructions of the manufacturer. Three parallels were made for each sample. The hypothalamus and medulla oblongata were transferred into a glass homogenizer, and 5–10 mL of pre-cooled PBS buffer (1:5 mass/volume ratio of tissue to PBS buffer is recommended) was added for thorough grinding, the prepared homogenate was centrifuged at 3500 r/min for 15 min, and then the supernatant was retained for the assay. This kit was specifically used to detect ACh and showed no obvious cross-reactivity with other similar substances. This kit was suitable for pan-species (general) in all tissues.

### 2.6. Total RNA Extraction, Reverse Transcription Analysis, and Quantitative Real-Time PCR

The mRNA expression of nicotinic acetylcholine receptor alpha 4 (α4 nAChR) in the hypothalamus and medulla oblongata and M3 muscarinic acetylcholine receptor (M3 mAChR) in the cecum were analyzed using quantitative real-time PCR. The primers were synthesized and provided by the Wuhan Sevier Biological Company (Wuhan, China). Total RNA was isolated from the cecum, cecal mucosa, and breast muscle employing a Trizol reagent, followed by reverse transcription utilizing the Prime Script RT Reagent Kit with gDNA Eraser from Takara (Dalian, China). Quantitative real-time PCR was conducted using TB Green Premix Ex Taq II (Takara) on a Light Cycler 96 PCR System to assess mRNA levels. Gene expression was measured using relative RT-PCR, with the primer sequences detailed in Table 2. The primers used are shown in Table 2. Relative quantification of gene expression was determined by 2^−ΔΔCt^ and Recombinant Glyceraldehyde-3-Phosphate Dehydrogenase (GAPDH) acted as the control gene.

### 2.7. Statistical Analysis

The data from the experiment were analyzed using a one-way ANOVA test, least significant difference (LSD) test, and a Duncan’s test, performed using SPSS 23.0 (SPSS Inc., Chicago, IL, USA). Mean values are presented in the tables, and the *p*-value was considered significant when *p* < 0.05. The figures in this study were generated using GraphPad Prism 8.0 (GraphPad Inc., San Diego, CA, USA). Replicate (n = 6) served as the experimental unit.

## 3. Results

### 3.1. Effects of Different Photoperiods on Growth Performance

The ADG, ADFI, and FE of broilers were evaluated to explore the effect of different photoperiods on the growth performance of broilers. As shown in Table 3, compared with the 12L:12D photoperiod, the 18L:6D and 24L:0D photoperiods have significantly enhanced effects on the ADG and ADFI of broilers in weeks 0–2, weeks 2–4, and weeks 0–4 (*p* < 0.05). At weeks 0–2, ADG increased by 17.33% and 19.33% exposed to the 18L:6D and the 24L:0D photoperiods, respectively, in comparison to that exposed to the 12L:12D photoperiod. Similarly, ADFI increased by 18.07% and 44.51% exposed to the 18L:6D and the 24L:0D photoperiods, respectively. At week 2–4, ADG (10.82% and 11.41%, respectively) and ADFI (20.89% and 33.95%, respectively) also showed significant increases exposed to the 18L:6D and the 24L:0D photoperiods in comparison to those exposed to the 12L:12D photoperiod. At week 0–4, ADG (12.53% and 13.58%, respectively) and ADFI (19.89% and 29.89%, respectively) were much higher exposed to the 18L:6D and the 24L:0D photoperiods than those exposed to the 12L:12D photoperiod. However, FE for subjects exposed to the 24L:0D photoperiod was significantly reduced in comparison to that exposed to the 12L:12D and 18L:6D photoperiods during the whole trial period (*p* < 0.05). These data suggest that long photoperiods improve the growth performance of broilers, but significantly decrease the FE of broilers.

### 3.2. Effects of Different Photoperiods on Glucose Metabolites in Broilers

We next investigated whether different photoperiods had effects on the glucose metabolism of the broilers. In Table 4, we show that the serum GLU, serum INS, and HOMA-IR of broilers are significantly enhanced in the 18L:6D photoperiod and the 24L:0D photoperiod compared with the 12L:12D photoperiod at week 2 (*p* < 0.05). Serum TG of broilers in the 24L:0D photoperiod was significantly increased than that in the 12L:12D photoperiod at week 2 (*p* < 0.05), but no significant difference was found between the 18L:6D photoperiod and the 12L:12D photoperiod (*p* > 0.05). At weeks 0–4, long photoperiods (18L:6D and 24L:0D) were associated with higher serum GLU, serum TG, serum INS, and HOMA-IR of broilers (*p* < 0.05). The results demonstrate that long photoperiods can induce glucose metabolism disorders, including severe insulin resistance.

### 3.3. Effects of Different Photoperiods on ACh and Its Relative ACh Receptor Modulation in Broilers

**Figure 1:**
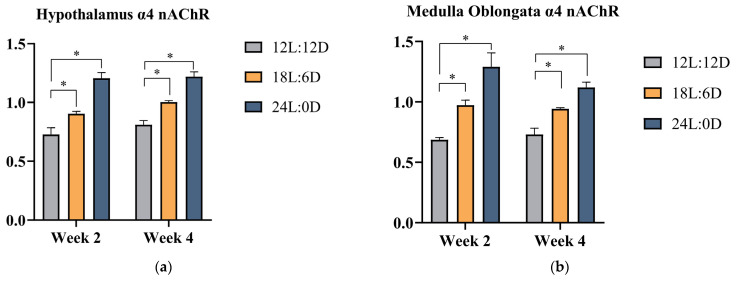
Effects of different photoperiods on the α4 nAChR mRNA expression levels of broilers. (**a**) Hypothalamus; (**b**) Medulla oblongata. Data are presented as Mean ± SEM. * *p* < 0.05. Abbreviations: α4 nAChR: nicotinic acetylcholine receptor alpha 4.

**Figure 2:**
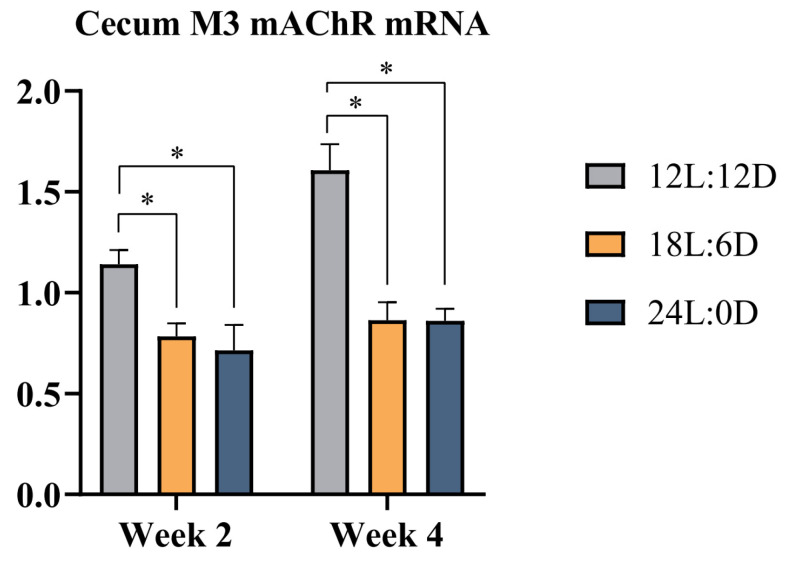
Effects of different photoperiods on the M3 mAChR mRNA expression levels in the cecum of broilers. Data are presented as Mean ± SEM. * *p* < 0.05. Abbreviations: M3 mAChR: M3 muscarinic acetylcholine receptor.

To validate the physiological activity results and further explore ACh and its relative ACh receptor modulation underlying different photoperiods, ACh concentrations, α4 nAChR mRNA expression levels in the hypothalamus and medulla oblongata, and M3 mAChR mRNA expression in the cecum were investigated. As depicted in Table 5, compared with the 12L:12D photoperiod, the ACh concentrations of broilers in the 18L:6D photoperiod and the 24L:0D photoperiod significantly increase at week 2 and week 4 (*p* < 0.05). Additionally, the ACh concentration in the 24L:0D photoperiod was statistically higher than that in the 18L:6D photoperiod (*p* < 0.05). As also shown in Figure 1, compared with the 12L:12D photoperiod, the 18L:6D photoperiod and the 24L:0D photoperiod result in significantly enhanced mRNA expression levels of α4 nAChR in the hypothalamus and medulla oblongata of broilers, with the highest evident in 24L:0D photoperiod at week 2 and week 4 (*p* < 0.05). Also depicted in Figure 2, the 18L:6D photoperiod and the 24L:0D photoperiod exhibit significantly lower levels of M3 mAChR mRNA expression in the cecum of broilers compared to the 12L:12D photoperiod (*p* < 0.05).

## 4. Discussion

In the current study, we identified the effects of different photoperiods on the growth performance, glucose metabolism, ACh, and its relative acetylcholine receptors in broilers. This is the first study to show that long photoperiods induced not only glucose metabolism disorders, but also ACh, α4 nAChR mRNA, and M3 mAChR mRNA expression modulation in broilers. By extending the duration of light in this study, the higher feed intake of broilers led to higher body weight gain, while resulting in significantly lower feed utilization.

In this study, extending the duration of light was found to increase the ADFI and ADG of broilers while reducing the FE. The results of this study demonstrate that both the 18L:6D photoperiod and the 24L:0D photoperiod are effective in increasing the growth rate of broilers. During the four-week trial period, there was a notable decrease in FE in the 24L:0D photoperiod. Similar results were also obtained by previous research that shows that long photoperiods enhance the growth rate and overall chicken production performance [28,29]. This finding aligns with previous studies that have reported faster growth rates and lower FE [30,31].

In this study, our findings also reveal that extended photoperiods can lead to glucose metabolism disorders in broilers. Glucose metabolism is the primary metabolic pathway to providing energy to the bodies. Serum glucose concentrations, as well as serum TG and serum INS concentrations, have become the basic indicators of glucose metabolism disorders since their abnormal elevation is caused by the impaired function of glucose utilization for cells induced by glucose metabolism disorders [32,33]. Moreover, previous studies have shown that glucose metabolism is disrupted when developing insulin resistance in broilers [17]. Our observation of the increase in serum glucose, TG, and INS concentrations in broilers when exposed to light for an extended duration provides compelling evidence supporting the idea that long photoperiods can lead to glucose metabolism disorders. Using the HOMA-IR index, this study revealed the insulin resistance resulting from broilers exposed to an extended duration of light, which suggests the reduced abilities of insulin-mediated glucose metabolism in broilers. The results of glucose metabolism disorders under different photoperiods in a previous study of redheaded buntings and mice are similar to our findings in this study. A previous study has shown that, compared with the 8L:16D photoperiod, the mRNA expression of *elovl6* was reduced in the 13L:11D photoperiod in redheaded buntings (*Emberiza bruniceps*), which might be related to insulin resistance [12]. Additionally, several reports have also shown that the glucose metabolism indexes, including serum glucose concentrations, glucose tolerance, and daily rhythms of glucose metabolism, can induce disorders by exposure to light at night in mice [13,14,15]. Herein, our results shed light on the disorder-inducing effects of long photoperiods on glucose metabolism in broilers.

In this study, the effects of extending the light duration were first found to regulate ACh and its relative acetylcholine receptors in broilers. ACh is the fundamental neurotransmitter of cholinergic neurons, which binds to muscarinic acetylcholine receptors (mAChRs) and nicotinic acetylcholine receptors (nAChRs) to play an important role in the nervous system [34]. α4 nAChR is a type of nAChR subunit, an important receptor in the nervous system, which is reported as a postsynaptic receptor of preganglionic nervous fibers and exists widely in chick brains to transmit nerve impulses [35,36,37]. Here, we identified that both ACh concentrations and α4 nAChR mRNA expression levels in the hypothalamus and medulla oblongata showed an increase in broilers exposed to light for an extended duration, indicating the improvement of postganglionic fibers in cholinergic neurons can be induced by exposure to long photoperiods. The presence of M3 mAChR in the epithelial cells of the intestine, an important receptor in the postganglionic fibers of parasympathetic nerves, suggests its involvement in regulating metabolic and feeding responses [38,39]. However, a significant decrease was also observed in M3 mAChR mRNA expression levels in the cecum under long-photoperiod conditions. These findings indicate that extended light exposure can increase ACh levels and the α4 nAChR expression of preganglionic nervous fibers in cholinergic neurons, but might inhibit cecum parasympathetic activities. Although there is no research on the parasympathetic activities in broilers because less attention has been paid to this activity, previous studies have shown light signal regulation exhibits higher parasympathetic activity during the night and higher sympathetic activity during the day in mammals [40,41].

In this study, the findings show abnormal cholinergic neuron activities and disorder glucose metabolism occur simultaneously, suggesting abnormal cholinergic neuron modulation might be closely related to glucose metabolism disorders in broilers exposed to light for an extended period. Previous research has reported that the cholinergic neuron system plays an indispensable role in regulating glucose metabolism through innervating pancreatic islets and libers [42,43]. In addition, the parasympathetic nervous system is an important part of the cholinergic nervous system. Previous research has also reported that the parasympathetic nerve system plays an indispensable role in regulating glucose metabolism through innervating pancreatic islets [18,19] and depending on the neuronal activities in mammals [44]. The parasympathetic nervous system mainly acts to promote digestion and accumulate energy [45,46], thus the decrease in FE in this study might be closely related to abnormal parasympathetic activities, too. Therefore, the effects and functions of the parasympathetic nervous system exposed to different photoperiods require further exploration. Moreover, the changes in feed conversion efficiency might also be attributed to glucose metabolism [17], suggesting the reduction in FE might be relevant to glucose metabolism disorders. In this study, we observed reduced FE accompanied by the increase in the serum GLU concentrations and insulin resistance, indicating the serum GLU of broilers could not be normally ingested by cells in the breast muscle or liver to synthesize glycogen to be deposited in the bodies, which also provided reliable evidence that glucose metabolism disorders are closely related to the lower FE of broilers exposed to long photoperiods. Moreover, feed intake also plays a vital role in regulating glucose metabolism in broilers. Previous research has shown that a lower feed intake can improve the fasting blood glucose of broilers [17]. Here, we observed the higher feed intake and glucose metabolism disorders of broilers exposed to long photoperiods, suggesting these two physiological reactions could be closely associated. Supplementary Material Table S1 shown the correlation of the FE, HOMA-IR, ACh, and M3 nAChR mRNA expressions. We will continue to conduct in-depth research on the effects of light exposure and glucose metabolism on broilers.

## 5. Conclusions

Extended light exposure has been found to enhance the growth rate of broilers while reducing feed efficiency. Furthermore, extended light exposure also induced glucose metabolism disorders, higher ACh concentrations, and α4 nAChR mRNA expression levels in the hypothalamus and medulla oblongata, and lower M3 mAChR mRNA expression levels in the cecum. Moreover, glucose metabolism disorder is closely related to the reduction in the health and quality of broilers, including woody breast severity [47], ascites syndrome [48], and tibial dyschondroplasia [49]. Therefore, it is crucial for the broiler industry to balance growth rates and the health and quality of broilers. These findings have important implications for future research on the role of the cholinergic neuron system and glucose metabolism in regulating the health of broilers exposed to extended-light photoperiods. In this study, the recommended lighting regime is 18L:6D to provide a better growth performance and health status for broilers.

## Figures and Tables

**Table 1:** Composition and nutrient levels of basic diets.

Items	5–7 d	8–22 d	23–33 d
Ingredient	Content (%)		
Corn	51.44	54.08	56.85
Soybean meal	40.21	36.82	33.86
Soybean oil	3.94	5.00	5.50
Limestone	1.00	0.85	0.90
CaHPO4	1.89	1.80	1.50
NaCl	0.30	0.30	0.30
DL-Methionine	0.21	0. 19	0.19
L-Lysine	0.36	0.32	0.30
L-Threonine	0.15	0. 14	0.10
Premix ^1^	0.50	0.50	0.50
Total	100	100	100
Nutrient levels ^2^			
ME/(Kcal/Kg)	2961	3038	3095
CP (%)	22.55	21.18	19.97
CF (%)	10.54	10.38	11.35
Ca (%)	0.94	0.85	0.79
AP (%)	0.43	0.41	0.35
Lysine (%)	1.46	1.34	1.25
Methionine (%)	0.54	0.51	0.49
Methionine + cysteine (%)	0.92	0.87	0.84

Abbreviations: ME, metabolizable energy; CP, crude protein; CF, crude fat; AP, available phosphorus. ^1^ Premix provided the following per kg of the diet: 5–7 d: vitamin A 12,000 IU, vitamin D3 5000 IU, vitamin E 80 mg, vitamin K3 3.2 mg, vitamin B1 3.2 mg, vitamin B2 8.6 mg, vitamin B6 4.3 mg, vitamin B12 17 μg, pantothenic acid calcium 20 mg, nicotinic acid 65 mg, folic acid 2.2 mg, biotin 0.22 mg, choline 1020 mg, Cu (CuSO_4_·5H_2_O) 16 mg, Fe (FeSO_4_·7H_2_O) 20 mg, Zn (ZnSO_4_·7H_2_O) 110 mg, Mn (MnSO_4_·H_2_O) 120 mg, Se (Na_2_SeO_3_) 0.3 mg, and I (KI) 1.25 mg; 8–22 d: vitamin A 10,000 IU, vitamin D3 4500 IU, vitamin E 65 mg, vitamin K3 3.0 mg, vitamin B1 2.5 mg, vitamin B2 6.5 mg, vitamin B6 3.2 mg, vitamin B12 17 μg, pantothenic acid calcium 18 mg, nicotinic acid 60 mg, folic acid 1.9 mg, biotin 0.18 mg, choline 1020 mg, Cu (CuSO_4_·5H_2_O) 16 mg, Fe (FeSO_4_·7H_2_O) 20 mg, Zn (ZnSO_4_·7H_2_O) 110 mg, Mn (MnSO_4_·H_2_O) 120 mg, Se (Na_2_SeO_3_) 0.3 mg, and I (KI) 1.25 mg; 23–33 d: vitamin A 9000 IU, vitamin D3 4000 IU, vitamin E 55 mg, vitamin K3 2.2 mg, vitamin B1 2.2 mg, vitamin B2 5.4 mg, vitamin B6 2.2 mg, vitamin B12 11 μg, pantothenic acid calcium 15 mg, nicotinic acid 45 mg, folic acid 1.6 mg, biotin 0.15 mg, choline 950 mg, Cu (CuSO_4_·5H_2_O) 16 mg, Fe (FeSO4·7H_2_O) 20 mg, Zn (ZnSO_4_·7H_2_O) 110 mg, Mn (MnSO_4_·H_2_O) 120 mg, Se (Na_2_SeO_3_) 0.3 mg, and I (KI) 1.25 mg. ^2^ ME determination was performed in the State Key Laboratory of Animal Nutrition and Feeding according to the bionic digestive operation manual 12L:12DS3, CP content determination was conducted by using a Kjeldahl nitrogen analyzer, and CF content determination was conducted by using a Soxhlet extractor. Other nutrient levels were calculated according to the Tables of Feed Composition and Nutritive Values in China (2022).

**Table 2:** Primer sequences of target and reference genes.

Gene	Primer Sequence (5′–3′)	Product Length (bp)	Gene Bank Number
GAPDH	F: ACTTTGGCATTGTGGAGGGT R: GGACGCTGGGATGATGTTCT	188	NM_204305.1
α4 nAChR	F: CGTCGCCAACATTTCGGATG R: GCTCTGAGGGGATTCGGATG	131	NM_001397350.1
M3 mAChR	F: GGAGACTGAGAAACGCACCA R: CTGCCGTTGCAGTTCATAGC	103	NM_001396514.1

Abbreviations: GAPDH: Recombinant Glyceraldehyde-3-Phosphate Dehydrogenase; α4 nAChR: nicotinic acetylcholine receptor alpha 4; M3 mAChR: M3 muscarinic acetylcholine receptor.

**Table 3:** Effects of different photoperiods on the growth performance of broilers.

Item	12L:12D	18L:6D	24L:0D	SEM	*p* Value
Weeks 0–2					
ADG/g	42.74 ^b^	50.10 ^a^	50.90 ^a^	0.93	<0.001
ADFI/g	45.07 ^c^	53.21 ^b^	65.10 ^a^	2.02	<0.001
FE	0.95 ^a^	0.94 ^a^	0.78 ^b^	0.02	<0.001
Weeks 2–4					
ADG/g	91.17 ^b^	101.01 ^a^	101.53 ^a^	1.32	<0.001
ADFI/g	113.55 ^c^	137.27 ^b^	152.10 ^a^	3.94	<0.001
FE	0.80 ^a^	0.74 ^b^	0.67 ^c^	0.01	<0.001
Weeks 0–4					
ADG/g	68.21 ^b^	76.76 ^a^	77.47 ^a^	1.06	<0.001
ADFI/g	78.15 ^c^	93.69 ^b^	101.50 ^a^	2.40	<0.001
FE	0.87 ^a^	0.82 ^b^	0.76 ^c^	0.01	<0.001

Abbreviations: ADG: average daily gain; ADFI: average daily feed intake; FE: feed efficiency. ^a–c^ Means within a row with different superscripts are significantly different (*p* < 0.05). SEM means standard error of the mean.

**Table 4:** Effects of different photoperiods on the serum glucose metabolites of broilers.

Item	12L:12D	18L:6D	24L:0D	SEM	*p* Value
Week 2					
Serum GLU/mmol/L	13.86 ^c^	15.27 ^b^	16.95 ^a^	0.47	0.002
Serum TG/mmol/L	2.78 ^b^	3.28 ^ab^	3.79 ^a^	0.17	0.015
Serum INS/mU/L	14.68 ^b^	17.60 ^b^	23.54 ^a^	1.39	0.002
HOMA-IR	9.04 ^c^	11.93 ^b^	17.75 ^a^	1.34	0.001
Week 4					
Serum GLU/mmol/L	13.27 ^c^	14.55 ^b^	15.90 ^a^	0.42	<0.05
Serum TG/mmol/L	3.10 ^b^	3.59 ^a^	3.77 ^a^	0.11	0.008
Serum INS/mU/L	15.37 ^c^	21.24 ^b^	24.72 ^a^	1.44	0.001
HOMA-IR	9.07 ^c^	13.73 ^b^	17.46 ^a^	1.25	0.007

Abbreviations: ADG: average daily gain; ADFI: average daily feed intake; FE: feed efficiency. ^a–c^ Means within a row with different superscripts are significantly different (*p* < 0.05). SEM means standard error of the mean.

**Table 5:** Effects of different photoperiods on the ACh concentrations of broilers.

Item	12L:12D	18L:6D	24L:0D	SEM	*p* Value
Week 2					
Hypothalamus/μg/mg	1.16 ^c^	1.66 ^b^	2.16 ^a^	0.16	0.008
Medulla oblongata/μg/mg	1.36 ^c^	1.83 ^b^	2.30 ^a^	0.15	0.008
Week 4					
Hypothalamus/μg/mg	1.09 ^c^	0.96 ^b^	1.45 ^a^	0.11	0.012
Medulla oblongata/μg/mg	0.76 ^c^	1.04 ^b^	1.38 ^a^	0.10	0.005

Abbreviations: ACh: acetylcholine. ^a–c^ Means within a row with different superscripts are significantly different (*p* < 0.05). SEM means standard error of the mean.

## Data Availability

The datasets used or analyzed during the study are available from the corresponding authors upon reasonable request.

## Supplementary Materials

The following supporting information can be downloaded at: https://www.mdpi.com/article/10.3390/ani14203003/s1, Table S1: The correlation of the FE, HOMA-IR, ACh, and M3 nAChR mRNA expressions.
