# Supplementary material for: Effects of Different Photoperiods on Growth Performance, Glucose Metabolism, Acetylcholine, and Its Relative Acetylcholine Receptor Modulation in Broiler Chickens
Source: Animals (Basel). 2024 Oct 17;14(20):3003. doi: 10.3390/ani14203003 (PMC11503876; doi:10.3390/ani14203003)
Supplement: Supplementary file 1 [file animals-14-03003-s001.zip › animals-3259354-supplementary.pdf]

animals

MDPI

Supplementary Material

A correlation of the FE, HOMA-IR, ACh, and M3 nAChR mRNA expressions was measured to additionally explain the relationship between the performance, glucose metabolism disorder and ACh signaling pathway. Correlation analysis was performed using Pearson linear correlation analysis. As shown in Table 1, FE was closely related to HOMA-IR, ACh, and M3 nAChR mRNA expressions, suggesting the reduction of feed efficiency might be induced by glucose metabolism disorder and parasympathetic activity inhibition.

Table S1. The correlation of the FE, HOMA-IR, ACh, and M3 nAChR mRNA expressions

**Items**	**HOMA-IR**		**Hypothalamus ACh**		**Medulla oblongata ACh**		**M3 nAChR mRNA**	
	Pearson coefficient	*P* value	Pearson coefficient	*P* value	Pearson coefficient	*P* value	Pearson coefficient	*P* value
Week 0-2 FE	-0.908	0.007	-0.815	0.007	-0.842	0.004	0.510	0.161
Week 2-4 FE	-0.922	<0.001	-0.827	0.006	-0.890	0.001	0.784	0.012

[1] Tables may have a footer.
